# Supplementary material for: The Effect of Hot Application Applied to the Breast with the Help of the Thera Pearl in the Postpartum Period on Mothers’ Milk Perception and Postpartum Breastfeeding Self-Efficacy: A Randomized Controlled Study
Source: Healthcare (Basel). 2024 May 8;12(10):968. doi: 10.3390/healthcare12100968 (PMC11121602; doi:10.3390/healthcare12100968)
Supplement: Supplementary file 1 [file healthcare-12-00968-s001.zip › healthcare-2914792-supplementary.pdf]

S1: The Effect of Hot Application Applied to the Breast with the Help of Thera Pearl in the Postpartum Period on Mothers' Milk Perception and Postpartum Breastfeeding Self-Efficacy: Randomized Controlled Study

PERSONAL INFORMATION FORM

1) What is your age?

1.20-25 2.26-30 3.31-35 4. 36 and over

2) Where do you live?

1.City 2.County 3. Villace

3) What is your educational background?

1. Primary school 2. Middle school 3. High school 4. University

4) What is your employment status?

1. I am working 2. I am not working

5) What is your family structure?

1. Nuclear Family 2. Extended Family

6) How would you evaluate your economic situation?

1. Income Less Than Expenses 2. Income Equals Expenditure 3. Income More Than Expenditure

Breastfeeding Information Form

Breastfeeding Observation Form to be Filled in the First 24 Hours After Birth

How full are the breasts?

Plump ( )   Not plump ( )

Was the baby breastfed in the first half hour?

Breastfed successfully ( )

breastfed with assistance ( )

Breastfeeding could not be achieved ( )

Has the baby been given formula?

Yes ( )   No ( )

If formula is given what is the reason?..............................

How does the baby latch on?

Good ( )

Middle ( )

Weak ( )

Has milk secretion started?

Comes After Milking By Hand ( )

Baby Breastfeeds Easily ( )

No Milk Release ( )

Postpartum Breastfeeding Self-Efficacy Scale

For each of these sentences, choose the statement that best describes how confident you are about breastfeeding. Answer by circling the number that closest to your feelings. There is no right or wrong answer.

1-Not at all sure 2-Very unsure 3-Sometimes sure 4-Sure 5-Very sure

1. I can always tell if my baby is getting enough milk.	1	2	3	4	5
2. I can always be successful in breastfeeding, as in other tasks.	1	2	3	4	5
3. I can breastfeed my baby at any time without additional formula.	1	2	3	4	5
4. I can always ensure that my baby latches on to the breast properly during breastfeeding.	1	2	3	4	5
5. I can always breastfeed to my satisfaction.	1	2	3	4	5
6. I can always breastfeed my baby, even if he cries.	1	2	3	4	5
7. I am always willing to breastfeed.	1	2	3	4	5
8. I can breastfeed my baby easily whenever I am with my family.	1	2	3	4	5
9. I am always happy to breastfeed.	1	2	3	4	5
10. I never mind if breastfeeding is time consuming.	1	2	3	4	5
11. I can wean my baby from the first breast I gave him before switching to the other breast.	1	2	3	4	5
12. I can breastfeed my baby at every meal.	1	2	3	4	5
13. I can always understand my baby's desire to suckle.	1	2	3	4	5
14. I can always tell when my baby wants to finish breastfeeding.	1	2	3	4	5

Insufficient Milk Perception Scale

This scale includes questions about your perceptions of your milk supply. Respond ‘yes’ or ‘no’ to the initial question about your current milk supply. Rate subsequent questions on a scale from 0 to 10, where 0 signifies no milk and 10 signifies an excessive supply. Scores closer to 0 suggest perceived insufficient milk, while scores closer to 10 suggest perceived sufficiency.

**1.**	Do you believe that you produce enough milk to feed your baby?	Yes ( )<br>No ( )
**2.**	My milk seems nutritious enough to feed my baby.	0 – 1 – 2 – 3 – 4 – 5 – 6 – 7 – 8 – 9 - 10
**3.**	My baby usually seems full after breastfeeding.	0 – 1 – 2 – 3 – 4 – 5 – 6 – 7 – 8 – 9 - 10
**4.**	My baby seems to love being breastfed.	0 – 1 – 2 – 3 – 4 – 5 – 6 – 7 – 8 – 9 - 10
**5.**	My milk contains all the nutrients my baby needs to develop.	0 – 1 – 2 – 3 – 4 – 5 – 6 – 7 – 8 – 9 - 10
**6.**	I seem to have enough milk in my breasts.	0 – 1 – 2 – 3 – 4 – 5 – 6 – 7 – 8 – 9 - 10
